# The variable monoaminergic outcomes of cleaner fish brains when facing different social and mutualistic contexts

**DOI:** 10.7717/peerj.4830

**Published:** 2018-05-24

**Authors:** Murilo S. de Abreu, João P.M. Messias, Per-Ove Thörnqvist, Svante Winberg, Marta C. Soares

**Affiliations:** 1 Programa de Pós-Graduação em Farmacologia, Universidade Federal de Santa Maria, Santa Maria, Brazil; 2 CIBIO, Centro de Investigação em Biodiversidade e Recursos Genéticos, Universidade do Porto, Portugal; 3 Department of Neuroscience, Uppsala Universitet, Sweden

**Keywords:** Serotonin, Dopamine, Cleanerfish, Mutualisms, Physical contact

## Abstract

The monoamines serotonin and dopamine are important neuromodulators present in the central nervous system, known to be active regulators of social behaviour in fish as in other vertebrates. Our aim was to investigate the region-specific brain monoaminergic differences arising when individual cleaners face a client (mutualistic context) compared to when they are introduced to another conspecific (conspecific context), and to understand the relevance of visual assessment compared to the impact of physical contact with any partner. We demonstrated that serotoninergic activity at the diencephalon responds mostly to the absence of physical contact with clients whereas cerebellar dopaminergic activity responds to actual cleaning engagement. We provide first insights on the brain’s monoaminergic (region-specific) response variations, involved in the expression of cleaner fishes’ mutualistic and conspecific behaviour. These results contribute to a better understanding of the monoaminergic activity in accordance to different socio-behavioural contexts.

## Introduction

The monoamines serotonin (5-hydroxytryptamine, 5-HT) and dopamine (3,4- dihydroxyphenethylamine, DA) are important monoaminergic neurotransmitters/neuromodulators at the central nervous system (CNS). These monoaminergic systems are evolutionarily well conserved and are the found in invertebrates as well as vertebrates (Adrio, Anadon & Rodriguez-Moldes, 1999; Azmitia, 2007). The monoaminergic modulation of behaviour (for instance by 5-HT and DA) depends on the nature of the G-protein-coupled receptors, to which monoamines bind to, and envolve several associated receptor types that act through different cell signaling mechanisms (Hoyer, Liebig & Rössler, 2005). The serotoninergic system is comprised by raphe nuclei and their projections to the preoptic area and the basal hypothalamus (Lillesaar, 2011; López & González, 2014; Maximino et al., 2013). On the other hand, dopaminergic innervation extends to the arcuate nucleus, homologous to the teleost’s nucleus lateralis tuberi, where DA is synthesized by DA’ producing neurons and released from their axons to the pituitary circulation (Holmqvist & Ekström, 1995; Pombal, El Manira & Grillner, 1997; Ben Jonathan & Hnasko, 2001).

Serotonin is important in the regulation of social behaviour in vertebrates (Fox, Ridgewell & Ashwin, 2009; Raleigh et al., 1991; Winberg et al., 1993). For instance, in humans, 5-HT dysfunction is associated with vulnerability to mood disorders (Fox, Ridgewell & Ashwin, 2009), such as depression and anxiety (Lesch & Mössner, 1998), and is also associated with antisocial (impulsive) behaviours and aggressive responses (Coccaro, Lee & Kavoussi, 2010). In teleost fish, is related to multiple brain functions, which also involve endocrine and stress responses (e.g., stress by chasing, stress coping) (Abreu et al., 2014; Puglisi-Allegra & Andolina, 2015). Similarly, DA is involved in the modulation of a wide variety of animal behavioural processes and cognition (for instance, in learning and reward/risk assessment, see Soares, 2017; Goodson, 2005). As part of the brain reward system, DA is an essential modulator in signalling the outcome of any action as either appetitive or aversive (Soares, 2017). Additionally, its key functions in associative learning (DeWitt, 2014) and behavioural reinforcement (Heimovics et al., 2009), DA also plays a role in risk assessment and decision making (Salamone & Correa, 2012; Schultz, 1998).

Knowledge regarding the proximate mechanisms mediating fish sociality, which includes both conspecific and interspecific (mutualistic) behaviour, is increasing but is still rather limited (Soares, 2017). Interspecific cleaning behaviour between fishes has long been a notorious example of mutualistic cooperation and communication in the aquatic (marine) environment (Côté, 2000). It is the case of the widely known Indo-pacific bluestreack cleaner wrasse (*Labroides dimidiatus)*, which removes ectoparasites, mucus, diseased or dead tissue from a huge variety of visitor fish species (known as clients) (Soares, 2017). These cleaners may engage in as many as 2,000 such interactions per day (Grutter, 1996), but interactions’ quality may vary from one client species to the next, and conflicts may arise, for instance: predatory clients may eat the cleaners (Bshary & Côté, 2008). But because these cleaners prefer to eat client derived mucus instead of parasites, which causes clients to interrupt and even punish cleaners, these are forced to reconcile and manipulate client decisions by providing physical contact (a form of tactile stimulation with their pelvic fins), which increases clients’ inspection durations, contributes to managing potential aggression by the predatory clients and reduces clients’ stress levels (Grutter, 2004; Bshary & Côté, 2008; Soares et al., 2011). Therefore, visual and contact-based communication between cleaners and clients are of key importance to the iterated maintenance and quality of these relationships.

Recent studies focusing on marine cleaning mutualisms have helped to explain the role of cleaners’ neuro-physiological state to their output behaviour (Soares, 2017). For instance, we now know that an increase in 5-HT availability makes cleaners more willing to interact and to provide tactile stimulation to clients (Paula et al., 2015). While blocking serotonin-mediated response affects their willingness to clean, contributes to increase aggressiveness toward smaller conspecifics (Paula et al., 2015) and seems to lower their ability to learn (Soares, Paula & Bshary, 2016). On the other hand, field experiments done aiming for the DAergic system revealed its decisive link to cleaners’ decision-making process, namely by seeking to interact more frequently but mostly in providing more tactile stimulation to clients, with the blockage of the D1 and D2-like receptors (Messias et al., 2016; Soares, Santos & Messias, 2017). In the lab, tests demonstrated a significant role of the DA agonists on cleaners’ ability to learn new tasks (Messias et al., 2016) but it also to modulate the motivational incentive that cleaners assign to client-derived cues (Soares et al., 2017b). Thus, at this point we know that these monoaminergic systems are crucially involved in how cleaners evaluate their social environment and, are significantly implicated in their behavioural output (Soares, 2017). However, the putative effects of different social contexts on 5-HT and DA activity in different brain regions are yet to be discovered. The aim of the present study is to examine the variations of 5-HT, DA and related metabolites, measured at different brain regions, within cleaners’ behavioural responses, facing different social and mutualistic contexts.

## Materials and Methods

### Animals and housing

Experiments were conducted at the fish housing facilities of the Oceanário de Lisboa (Lisbon, Portugal). A stock population of 53, adult, Indo-Pacific bluestreak cleaner wrasse, *Labroides dimidiatus,* (∼95% females) and 10 adult blond naso tang *Naso elegans* (family Acanthuridae, hereafter referred as clients) were used in the study, all imported to Portugal by a local distributor (Tropical Marine Centre, Lisbon, Portugal). Total length and total weight of cleaner wrasses *(L. dimidiatus*) ranged from 5.2 to 7.2 cm (mean ± SD 6.4 ± 0.6 cm) and 1.2 to 4.7 g (2.4 ± 0.7 g), and tangs *N. elegans* ranged from 9 to 15 cm (11.6 ± 1.9 cm) and 10.2 to 49.6 g (25.2 ± 12.5 g). Cleaners were kept alone in 50 × 40 × 40 cm aquaria while tangs were kept in stock aquaria of 100 × 40 × 40 cm, in groups of 5–10 individuals. All fish were left to acclimatize a minimum of 15 days before the start of treatments. Animals were fed twice a day (morning and afternoon): cleaners mostly on mysis shrimp, and clients on a mixture of fin-cut vegetables (broccoli and carrots) with mashed shrimp and mussels. All aquaria were combined in a flow through system that pumped water from a larger sump (150 × 50 × 40 cm) that served as a mechanical and biological filter. Nitrite concentration was kept to very low levels (always below 0.3 mg/l). Each tank contained an air supply and a commercial aquarium heater (125 W, Eheim, Jäger). PVC pipes (15–20 cm long; 20 cm diameter) served as shelter for the fish. Animals were kept in tropical conditions: water temperature between 24 and 26 °C and 12-h photoperiod (6 AM–6 PM). Experiments were carried out in the individual smaller tanks (50 × 40 × 40 cm). This study was approved by the Portuguese Veterinary Office (Direcção Geral de Veterinária, license # 0420/000/000/2009) and was carried out in accordance with the approved guidelines.

### Experimental design and sampling

On each experimental day, one of the following treatments was randomly allocated to each subject cleaner wrasses’ aquarium: (a) group A (conspecific, *L. dimidiatus*, *n* = 10), (b) group B (client, *N. elegans, n* = 11), (c) group C (conspecific inside another smaller aquarium, *n* = 12), (d) group D (client inside another smaller aquarium, *n* = 10) and (e) group E (white ball, about 5 cm in diameter, which stayed at the bottom, completely sessile; Soares et al., 2017a, *n* = 10) (see schematic drawing at the Supplemental Information). Experimental aquaria were divided by opaque partitions that prevented subject client fish from observing other individuals (inside other aquaria) during experiments. Behavioural trials started when treatments were introduced to each subject cleaner. Behaviour was then videotaped for the next 60 min while the experimenter left the room (see section behavioural analyses below). At the end of the experiments, each subject cleaner was captured and immediately sacrificed with anesthetic (MS222; Pharmaq, Overhalla, Norway; 1,000 mg/L) that was added to the water, working rapidly to anesthetize the fish and finalized with the complete transection of the spinal cord. The brain was immediately dissected (without buffering) under a stereoscope (Stemi 2000; Zeiss, Oberkochen, Germany) into five macro-areas: forebrain (olfactive bulbs + telencephalon), diencephalon, optic tectum, cerebellum and brain stem, with the duration of the dissection never exceeding 5 min. Major brain areas were frozen with dry ice and then stored at −80 °C. Animal procedures used in this study were approved by the Portuguese Veterinary Office (Direcção Geral de Veterinária, license # 0420/000/000/2009) and were carried out in accordance with the approved guidelines.

### Quantification of monoamines by high performance liquid chromatography with electrochemical detection (HPLC-EC)

The macroareas were homogenized in 4% (w/v) ice-cold perchloric acid containing 100 ng/ml 3,4-dihydroxybenzylamine (DHBA, the internal standard) using a Sonifier cell disruptor B-30 (Branson Ultrasonics, Danbury, CT, USA) and were immediately placed on dry ice. Subsequently, the homogenized samples were thawed and centrifuged at 21,000× g for 10 min at 4 °C. The supernatant was used for high performance liquid chromatography with electrochemical detection (HPLC-EC), analyzing the monoamines DA and 5-HT (5-hydroxytryptamine) the DA metabolite DOPAC (3,4-dihydroxyphenylacetic acid), and the 5-HT metabolite 5-HIAA (5-hydroxy indole acetic acid), as described by Øverli, Harris & Winberg (1999). In brief, the HPLC–EC system consisted of a solvent delivery system model 582 (ESA, Bedford, MA, USA), an auto injector Midas type 830 (SparkHolland, Emmen, the Netherlands), a reverse phase column (Reprosil-Pur C18-AQ 3 µm, 100 mm × 4 mm column, Dr. Maisch HPLC GmbH, Ammerbuch-Entringen, Germany) kept at 40 °C and an ESA 5200 Coulochem II EC detector (ESA, Bedford, MA, USA) with two electrodes at reducing and oxidizing potentials of −40 mV and +320 mV, respectively. A guarding electrode with a potential of +450 mV was employed before the analytical electrodes to oxidize any contaminants. The mobile phase consisted of 75 mM sodium phosphate, 1.4 mM sodium octyl sulphate and 10 µM EDTA indeionized water containing 7% acetonitrile brought to pH 3.1 with phosphoric acid. Samples were quantified by comparison with standard solutions of known concentrations. To correct for quantified DHBA was used as an internal standard using HPLC software ClarityTM (DataApex Ltd., Prague, Czech Republic). The ratios of 5-HIAA/5-HT and DOPAC/DA were calculated and used as an index of 5-HTergic and DAergic activity, respectively. For normalization of brain monoamine levels, brain protein weights were determined with Bicinchoninic acid protein determination (Sigma–Aldrich, Sweden) according to the manufacturer’s instructions. The assay was read on Labsystems multiskan 352 plate reader (Labsystems, Thermo Fisher Scientific, Waltham, MA, USA) wavelength of 570 nm.

### Behavioural analyses

During each video analysis, we recorded: (1) the number and duration (in seconds) of a cleaning inspection toward each client or other cleaner; (2) the frequency and duration of tactile stimulation provided (where a cleaner touches, with its fins, the body of the client and no feeding is involved Bshary & Würth, 2001); (3) the number of jolts by clients (cleaners sometimes take bites to which the clients respond with a short body jolt, that is usually associated with cheating by cleaners Bshary & Grutter, 2002; Soares et al., 2008); (4) number and duration (in seconds) of chases where the subject (focal individual) rapidly advanced towards the other conspecific; and finally (5) number of bites (punctual hit by cleaner’s mouth to clients’ body, to which the clients respond with a short body jolt. In the conspecific context, although we tried to match the sizes of the individuals, this was not always possible. Thus, the incidence of chases by the subject could be due to size differences (a.k.a., intruder is larger than the resident) or to sex differences, which we could not control for. For focal individuals introduced to either a conspecific or client inside another smaller aquarium, the larger aquaria were visually divided in two, so that we could quantify cleaners time spent swimming in each section. Videos were blindly scored, and analysed for the entire duration (60 min), by one single observer.

### Statistical analyses

Data were analysed using non-parametric tests because the assumptions for parametric testing were not met. Mann–Whitney *U*-tests were used to analyse behavioural measures. Kruskal–Wallis ANOVAs were performed to detect differences between treatments (five groups) for each brain area followed by Dunn’s Post-Hoc tests, which already include a Bonferroni adjustment to account for multiple comparisons, as to compare each treatment against the control group. Finally, relationships within and between behavioural measures, and clients’ brain monoaminergic levels were examined using Spearman correlation coefficients. We then proceeded to correct our *p* values by applying the Benjamini–Hochberg false discovery rate correction (Benjamini & Hochberg, 1995), reporting in the text just the correlations that remained significant.

## Results

### Cleaners behaviour

There were differences in the behavioural response of cleaners across our five experimental treatments. But solely on two of these experimental treatments did behavioural interactions occurred cleaners interacted mostly with clients (group A) but also with conspecifics (group B) when these were accessible (Table 1). To further confirm whether the distinction between these two groups (group A vs. B) was being consistently expressed, we compared each behavioural measure. The frequency of cleaning interactions was significantly higher when cleaners were introduced to clients compared to those introduced to a conspecific (Mann Whitney *U* test, *U* = 0, *n*1 = 8, *n*2 = 10, *p* < 0.0001), and the same occurred with the mean interaction time (Mann Whitney *U* test, *U* = 6.5, *n*1 = 8, *n*2 = 10, *p* = 0.0013), the frequency of cleaning bites (Mann Whitney *U* test, *U* = 0, *n*1 = 8, *n*2 = 10, *p* < 0.0001), the frequency of client jolts (Mann Whitney *U* test, *U* = 10, *n*1 = 10, *n*2 = 10, *p* = 0.0007) (Table 1). For all remaining behavioural measures (proportion of cleaning interactions in which tactile stimulation was provided, proportion of time providing tactile stimulation, and incidence of chases—Mann Whitney *U* tests) did not differ in contact with a conspecific or client (Table 1). Cleaners that were solely in visual contact with a client or a conspecific (because these were inside smaller aquaria) did not spend more time near the aquaria (Mann Whitney *U* test; Table 1). Finally, cleaners did not approach or had any contact with the ball (hereafter referred as ball control).

**Table 1:** Frequency of observed behavioural measures for each experimental treatment. These include: number of interactions; average interaction time; cleaning bites; proportion of interactions with tactile stimulation; proportion of time spent providing tactile stimulation; frequency client jolts/100 s; and incidence of chases, for groups A and B; and percentage time spent near the smaller aquarium for groups C and D. Mean ± Standard Error (SEM) are provided for each behavioural measure.

**Behaviour**	**Experimental treatments**		
	***Group A*** (*n* = 8)	***Group B*** (*n* = 10)	**Mann–Whitney *U* test**
Number of interactions	0.25 ± 0.16	22.1 ± 2.83	*P* < 0.0001^*^
Average interaction time	1 ± 0.75	6.68 ± 1.8	*P* = 0.0013^*^
Cleaning bites	0.12 ± 0.12	50.9 ± 5.64	*P* < 0.0001^*^
Proportion of interactions with tactile stimulation	0.62 ± 0.5	0.78 ± 0.1	*P* = 0.0623
Proportion of time spent providing tactile stimulation	0.15 ± 0.1	0.1 ± 0.02	*P* = 0.1329
Frequency client jolts/100 s	0	2.29 ± 0.79	*P* = 0.0007^*^
Incidence of chases	0.75 ± 0.53	1.1 ± 0.5	*P* = 0.4667
	***Group C*** (*n* = 12)	***Group D*** (*n* = 10)	**Mann–Whitney *U* test**
Percentage time spent near the smaller aquarium	0.25 ± 0.05	0.17 ± 0.08	*P* = 0.2498

**Notes.**

* Statistically significant result.

### Cleaners brain monoamines

#### Brain 5-HT and 5-HIAA levels, and 5-HIAA/5-HT ratios

**Figure 1:**
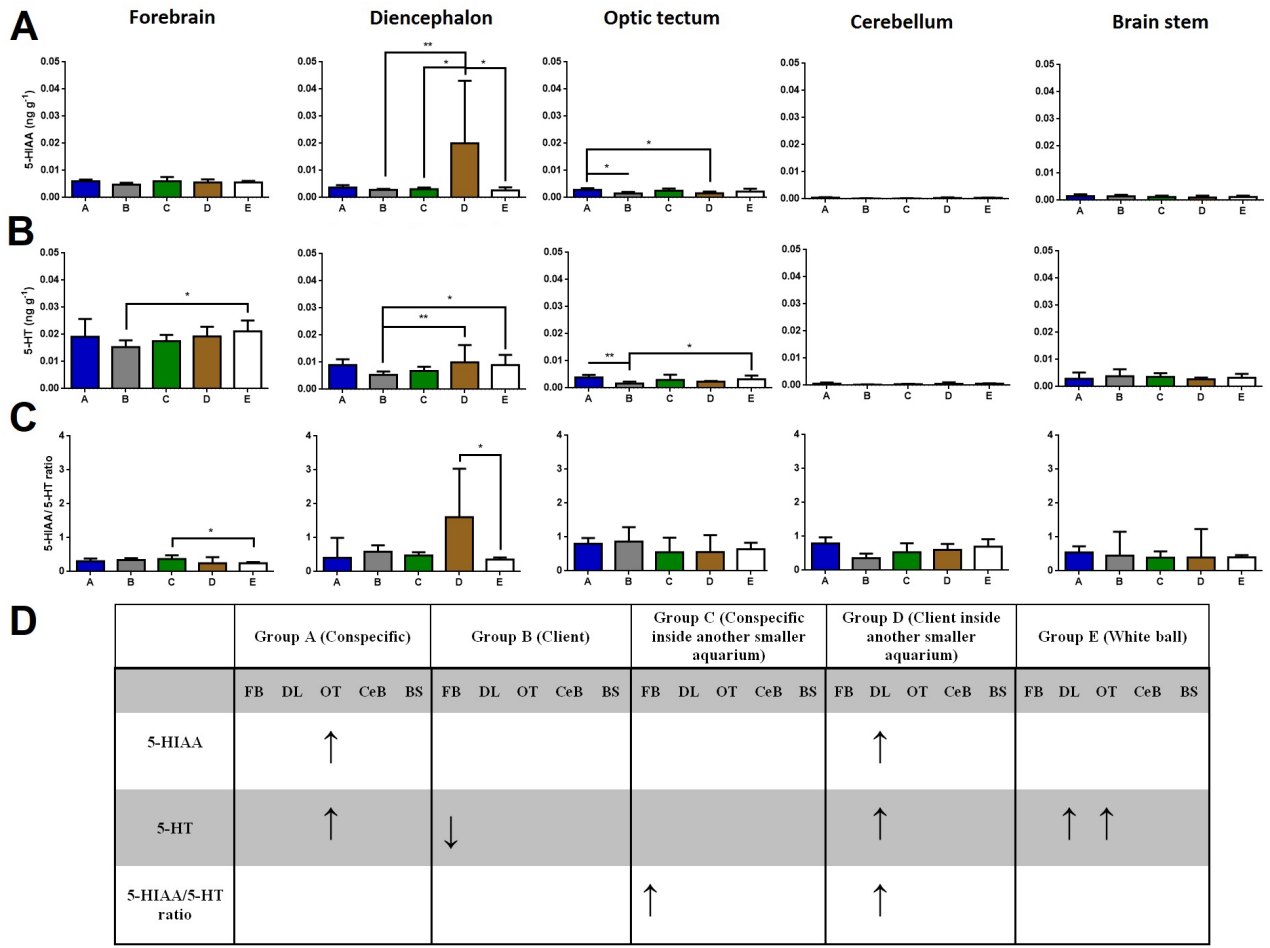
5-HIAA, 5-HT, and 5-HIAA/5-HT ratio in different brain areas, and different treatments. (A) 5-HIAA levels, in the forebrain (*K* = 7.657; *p* = 0.105), diencephalon (*K* = 14.78; *p* = 0.0052), optic tectum (*K* = 13.95; *p* = 0.0074), cerebellum (*K* = 11.85; *p* = 0.0185), brain stem (*K* = 2.809; *p* = 0.5903); (B) 5-HT levels, in the forebrain (*K* = 11.05; *p* = 0.026), diencephalon (*K* = 17.55; *p* = 0.0015), optic tectum (*K* = 17.9; *p* = 0.0013), cerebellum (*K* = 10.09; *p* = 0.0389), brain stem (*K* = 3.821; *p* = 0.4307); (C) 5-HIAA/5-HT ratio, in the forebrain (*K* = 10.73; *p* = 0.0298), diencephalon (*K* = 9.69; *p* = 0.0460), optic tectum (*K* = 6.996; *p* = 0.1361), cerebellum (*K* = 5.843; *p* = 0.2112), and brain stem (*K* = 2.47; *p* = 0.6500); (D) significant changes in different social contexts. Medians and interquartile ranges are shown. Significant values are shown above bars: * <0.05; ** <0.01; and refer to Dunn’s Post-Hoc tests.

Regarding brain 5-HIAA levels, cleaners introduced to a client inside another smaller aquarium increased 5-HIAA in the diencephalon compared to cleaners in full contact with a client, to cleaners introduced to a conspecific inside another smaller aquarium, and to cleaners introduced to a ball control (Dunn’s Post Hoc Test, *p* = 0.0052, Fig. 1). Furthermore, cleaners in contact with a conspecific increased 5-HIAA levels in the optic tectum compared to cleaners in contact with a client, and to cleaners introduced to a client inside another smaller aquarium (Dunn’s Post Hoc Test, *p* = 0.0074, Fig. 1).

Cleaners in contact with a client decreased in forebrains’ 5-HT levels compared to cleaners introduced to a ball control (Dunn’s Post Hoc Test, *p* = 0.026, Fig. 1). When cleaners were in contact with a client inside another smaller aquarium or introduced to a ball control, diencephalic 5-HT concentrations were elevated compared to cleaners in contact with a conspecific (Dunn’s Post Hoc Test, *p* = 0.0015, Fig. 1). Cleaners in contact with a conspecific or introduced to a ball control, showed elevated 5-HT levels in the optic tectum compared to cleaners in contact with a client (Dunn’s Post Hoc Test, *p* = 0.0013, Fig. 1).

Cleaners introduced to a conspecific inside another smaller aquarium showed higher 5-HIAA/5-HT ratios in the forebrain than cleaners introduced to a ball control (Dunn’s Post Hoc Test, *p* = 0.0298, Fig. 1), and also higher 5-HIAA/5-HT ratios in the diencephalon than cleaners in contact with a client inside another smaller aquarium (Dunn’s Post-Hoc Test, *p* = 0.0460, Fig. 1).

#### Brain DA and DOPAC concentrations, and DOPAC/DA ratios

**Figure 2:**
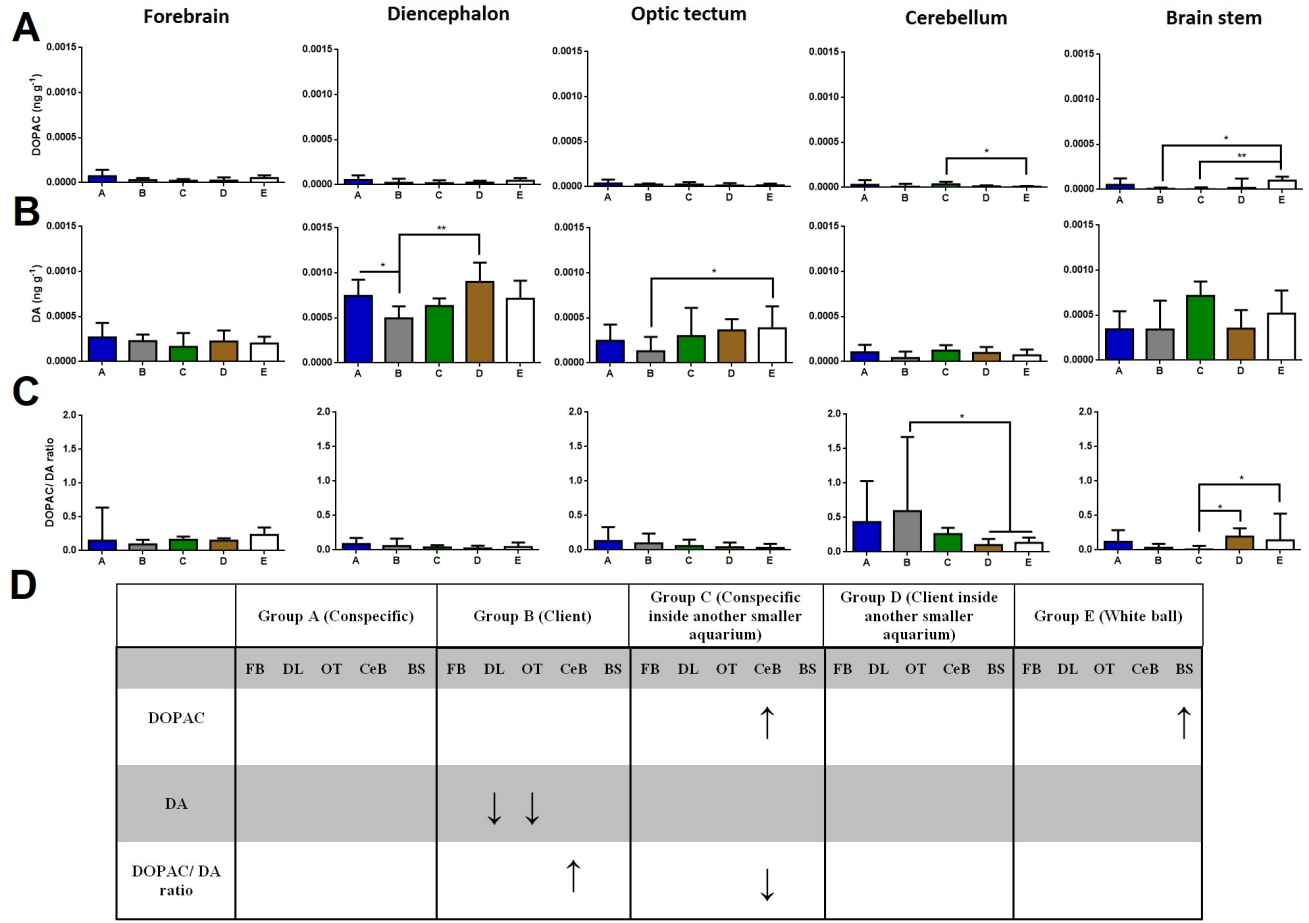
DOPAC, DA, and DOPAC/DA ratio in different brain areas, and different treatments. (A) DOPAC levels, in the forebrain (*K* = 8.901; *p* = 0.0636), diencephalon (*K* = 7.620; *p* = 0.1065), optic tectum (*K* = 2.115; *p* = 0.7146), cerebellum (*K* = 13.51; *p* = 0.0090), brain stem (*K* = 16.7; *p* = 0.0022); (B) DA levels, in the forebrain (*K* = 4.223; *p* = 0.3767), diencephalon (*K* = 16.18; *p* = 0.0028), optic tectum (*K* = 11.21; *p* = 0.0243), cerebellum (*K* = 8.428; *p* = 0.0771), brain stem (*K* = 6.268; *p* = 0.18); (C) DOPAC/DA ratio, in the forebrain (*K* = 5.328; *p* = 0.2553), diencephalon (*K* = 5.67; *p* = 0.2252), optic tectum (*K* = 4.483; *p* = 0.3446), cerebellum (*K* = 15.17; *p* = 0.0044), brain stem (*K* = 15.85; *p* = 0.0032); (D) significant changes in different social contexts. Medians and interquartile ranges are shown. Significant values are shown above bars: * <0.05; ** <0.01; and refer to Dunn’s Post-Hoc tests.

Regarding DOPAC brain levels, cleaners introduced to a conspecific inside another smaller aquarium increased its levels at the cerebellum compared to the ball control (Dunn’s Post-Hoc Test, *p* = 0.009; Fig. 2). When in contact with a client and introduced to a conspecific inside another smaller aquarium, DOPAC levels decreased in the brain stem compared to control-ball (Dunn’s Post-Hoc Test, *p* = 0.0022; Fig. 2).

Cleaners in contact with a client decreased in DA levels at the diencephalon compared to those in contact with conspecific or to those introduced to a client inside an aquarium (Dunn’s Post-Hoc Test, *p* = 0.0028; Fig. 2). Moreover, cleaners in contact with client decreased in DA levels at the optic tectum, compared to the ball control (Dunn’s Post-Hoc Test, *p* = 0.0243; Fig. 2).

Cleaners in contact with a client increased in DOPAC/DA ratios in the cerebellum compared to those introduced to a client inside an aquarium and to the ball control (Dunn’s Post-Hoc Test, *p* = 0.0044; Fig. 2). On the other hand, when in sole visual contact with other conspecifics, the DOPAC/DA ratios of cleaners decreased at the brain stem when compared to those in sole visual contact to clients and to controls (Dunn’s Post-Hoc test, *p* = 0.0032; Fig. 2).

### Relationship between monoamines and behaviour

The relationship between 5-HT and behavioural measures were solely significant when cleaners were in contact with conspecifics. Indeed, we found significant negative relationships between: (a) number of cleaning interactions and 5-HT in all brain areas (*P* < 0.0001, Table 2), (b) mean interaction time and 5-HT in all brain areas (*P* < 0.0001, Table 2), (c) frequency of cleaning bites and 5-HT in all brain areas (*P* < 0.0001, Table 2), (d) proportion of cleaning interaction in which tactile stimulation was provided and 5-HT in all brain areas (*P* < 0.0001, Table 2), and (e) proportion of time proving tactile stimulation and 5-HT in all brain areas (*P* < 0.0001, Table 2).

**Table 2:** Correlations (Spearman correlation coefficients) between each behavioural measure and brain serotonin levels in different brain macro-areas: forebrain, diencephalon, optic tectum, cerebellum and brain stem; for two experimental treatments: conspecific introduced to cleaner and client introduced to cleaner. Significant correlations are highlighted in bold and marked with * for *p* < 0.05, that correlation was true for the false discovery rate test.

Behaviour	Brain macro-areas				
	Forebrain	Diencephalon	Optic tectum	Cerebellum	Brain stem
Conspecific *N* = 8					
Number of interactions	*r* = − 0.6299* P < 0.0001	*r* = − 0.5040* P < 0.0001	*r* = − 0.6299* P < 0.0001	*r* = − 0.6299* P < 0.0001	*r* = − 0.7559* *P* < 0.0001
Average interaction time	*r* = − 0.6547* P < 0.0001	*r* = − 0.5144* P < 0.0001	*r* = − 0.5923* P < 0.0001	*r* = − 0.5923* P < 0.0001	*r* = − 0.7638* P < 0.0001
Cleaning bites	*r* = − 0.5774* P < 0.0001	*r* = − 0.4124* P < 0.0001	*r* = − 0.2474* P < 0.0001	*r* = − 0.2474* P < 0.0001	*r* = − 0.5774* P < 0.0001
Proportion of interactions with tactile stimulation	*r* = − 0.6547* P < 0.0001	*r* = − 0.5144* P < 0.0001	*r* = − 0.5923* P < 0.0001	*r* = − 0.5923* P < 0.0001	*r* = − 0.7638* P < 0.0001
Proportion of time spent providing tactile stimulation	*r* = − 0.6547* P < 0.0001	*r* = − 0.5144* P < 0.0001	*r* = − 0.5923* P < 0.0001	*r* = − 0.5923* P < 0.0001	*r* = − 0.7638* P < 0.0001
Incidence of chases	*r* = 0.1559 *P* = 0.75	*r* = 0.7326 *P* = 0.0714	*r* = 0.5455 *P* = 0.1786	*r* = 0.4364 *P* = 0.3214	*r* = 0.4053 *P* = 0.3571
Client *N* = 10					
Number of interactions	*r* = − 0.2317 *P* = 0.5060	*r* = − 0.7012* P = 0.0261	*r* = 0.2622 *P* = 0.4618	*r* = − 0.01220 *P* = 0.9665	*r* = − 0.3537 *P* = 0.3056
Average interaction time	*r* = − 0.2364 *P* = 0.5135	*r* = 0.2485 *P* = 0.4918	*r* = − 0.6606* P = 0.0438	*r* = − 0.3455 *P* = 0.3304	*r* = − 0.7212* P = 0.0234
Cleaning bites	*r* = − 0.2614 *P* = 0.4527	*r* = − 0.4499 *P* = 0.1864	*r* = − 0.1581 *P* = 0.6491	*r* = − 0.1945 *P* = 0.6491	*r* = − 0.6201 *P* = 0.0576
Proportion of interactions with tactile stimulation	*r* = 0.05839 *P* = 0.8775	*r* = − 0.1752 *P* = 0.4882	*r* = 0.4671 *P* = 0.1747	*r* = 0.5579 *P* = 0.0984	*r* = 0.6487* P = 0.0492
Proportion of time spent providing tactile stimulation	*r* = − 0.3576 *P* = 0.3129	*r* = 0.1758 *P* = 0.6321	*r* = − 0.1758 *P* = 0.6321	*r* = − 0.3212 *P* = 0.3679	*r* = − 0.1152 *P* = 0.7589
Frequency client jolts/100 s	*r* = 0.1702 *P* = 0.6371	*r* = 0.5775 *P* = 0.0851	*r* = − 0.7112* P = 0.0240	*r* = 0.03040 *P* = 0.9382	*r* = − 0.07295 *P* = 0.8298

Similarly, significant negative relationships between DOPAC/DA brain levels and cleaners’ behavioural response were only found when cleaners were introduced to other conspecifics, in terms of: (a) frequency of incidence of chases with DOPAC/DA at the diencephalon (*P* < 0.0001) and (b) frequency of cleaning bites with DOPAC/DA at the forebrain (*P* < 0.0001) and the cerebellum (*P* < 0.0001) (see Table 3).

**Table 3:** Correlations (Spearman correlation coefficients) between each behavioural measure and brain dopamine levels in different brain macro-areas: forebrain, diencephalon, optic tectum, cerebellum, and brain stem; for two experimental treatments: conspecific introduced to cleaner and client introduced to cleaner. Significant correlations are highlighted in bold and marked with * for *p* < 0.05, that correlation was true for the false discovery rate test.

Behaviour	Brain macro-areas				
	Forebrain	Diencephalon	Optic tectum	Cerebellum	Brain stem
Conspecific *N* = 8					
Number of interactions	*r* = 0.1260 *P* = 0.6429	*r* = 0.5040 *P* = 0.2857	*r* = 0.5040 *P* = 0.2857	*r* = − 0.1260 *P* = 0.2857	*r* = 0.3780 *P* = 0.4286
Average interaction time	*r* = 0.09352 *P* = 0.6786	*r* = 0.4520 *P* = 0.2857	*r* = 0.4520 *P* = 0.2857	*r* = − 0.1559 *P* = 0.3214	*r* = 0.3429 *P* = 0.3929
Cleaning bites	*r* = − 0.08248* P < 0.0001	*r* = 0.08248 *P* = 0.2500	*r* = 0.08248 *P* = 0.2500	*r* = − 0.2474* P < 0.0001	*r* = 0.08248 *P* = 0.2500
Proportion of interactions with tactile stimulation	***r*** = 0.09352 ***P*** = 0.6786	***r*** = 0.4520 ***P*** = 0.2857	***r*** = 0.4520 ***P*** = 0.2857	***r*** = − 0.1559 ***P*** = 0.3214	***r*** = 0.3429 ***P*** = 0.3929
Proportion of time spent providing tactile stimulation	*r* = 0.09352 *P* = 0.6786	*r* = 0.4520 *P* = 0.2857	*r* = 0.4520 *P* = 0.2857	*r* = − 0.1559 *P* = 0.3214	*r* = 0.3429 *P* = 0.3929
Incidence of chases	*r* = 0.07793 *P* = 0.6429	*r* = − 0.5455* P < 0.0001	*r* = 0.04676 *P* = 0.5714	*r* = 0.2962 *P* = 0.5	*r* = − 0.265 *P* = 0.1786
Client *N* = 10					
Number of interactions	*r* = 0.1463 *P* = 0.6864	*r* = 0.1524 *P* = 0.6743	*r* = 0.2744 *P* = 0.4403	*r* = − 0.1037 *P* = 0.7637	*r* = − 0.1341 *P* = 0.6997
Average interaction time	*r* = − 0.2970 *P* = 0.4069	*r* = 0.006061 *P* > 0.9999	*r* = − 0.06667 *P* = 0.8651	*r* = − 0.01818 *P* = 0.9730	*r* = 0.5394 *P* = 0.1139
Cleaning bites	*r* = 0.1033 *P* = 0.7773	*r* = 0.2736 *P* = 0.4412	*r* = 0.2918 *P* = 0.4112	*r* = − 0.2553 *P* = 0.4622	*r* = 0.09119 *P* = 0.8037
Proportion of interactions with tactile stimulation	*r* = 0.08434 *P* = 0.8177	*r* = − 0.03892 *P* = 0.7624	*r* = 0.2076 *P* = 0.5610	*r* = − 0.2141 *P* = 0.4202	*r* = − 0.5644 *P* = 0.0542
Proportion of time spent providing tactile stimulation	*r* = − 0.2727 *P* = 0.4483	*r* = − 0.3333 *P* = 0.3487	*r* = − 0.2606 *P* = 0.4697	*r* = − 0.3333 *P* = 0.3487	*r* = 0.1273 *P* = 0.733
Frequency client jolts/100 s	*r* = − 0.07903 *P* = 0.8177	*r* = − 0.06687 *P* = 0.8447	*r* = − 0.01216 *P* = 0.9655	*r* = − 0.1337 *P* = 0.699	*r* = 0.3647 *P* = 0.2985

## Discussion

Overall, this study provides first insights on the response of the monoaminergic brain (region specific) system linked to the expression of mutualistic behaviour in cleaners: the diencephalic 5-HTergic system responding mostly to the absence of physical engagement with clients and the cerebellum DAergic function responding significantly to cleaning interactions. Behaviourally, we found that cleaners were mostly engaging in cleaning interactions. Surprisingly, they were not behaving agonistically with conspecific partners and were not interacting in the control situation.

When kept in treatment groups that solely allowed for visual contact with partners, time spent near clients or conspecifics did not differ significantly. Specifically, we found that DOPAC/DA ratios increased in the cerebellum of cleaners that could interact freely with clients, compared to cleaners solely in visual contact with clients, as well as those in the control situation. In contrast, a decrease of DOPAC/DA levels were found at the brain stem of cleaners in exclusive visual contact with clients and in the control situation. Seemingly, it was also the inability interact (groups C and D, Fig. 1) that triggered a response of the cleaners’ 5HTergic system (5-HIAA/5-HT ratio) at the forebrain and diencephalon.

### A closer look to cleaners behavioural output

As expected, cleaners interacted more with clients than with the conspecifics. In natural conditions, these cleaners are acknowledged to inspect an average of 2297 fish clients per day (Grutter, 1996), a value that certainly extends beyond the number of interactions they have with conspecifics (M Soares, pers. obs., 2009, 2010, 2011). However, 5-HIAA/5-HT and DOPAC/DA ratios correlated significantly with behavioural measures exclusively when cleaners were in contact with conspecifics. This may have occurred due to the specific settings of our lab conditions in the conspecific context: because cleaners were exposed to an unfamiliar individual, we may have increased the probability of confrontations. It was perhaps this insecurity-matching contextual situation together with a lower overall level of interactions which generated a series of negative relationships.

### Dopaminergic change in accordance to social context

Cerebellum levels of DOPAC/DA ratios increased in cleaners left in contact with clients. Indeed, the teleost cerebellum and associated brainstem circuitry is strongly implicated in cognitive and emotional functions, namely in those linked to associative learning and memory processes (Rodriguez-Ortiz, 2005). It is through associative learning that animals learn to anticipate the occurrence of important behavioural outcomes, and perhaps how most of cleaners’ behavioural tactics are acquired (Soares et al., 2017a). Brain DA’ neurotransmission is crucial to potentiate the learning process in cleaners (see Messias et al., 2016), particularly in situations of novelty (Soares et al., 2017b). The cerebellar neural circuits have long been implicated in the coding of first-error teaching signals in which learning is based (see Houk, Buckingham & Barto, 1996; Ito, 1989; Kawato & Gomi, 1992; Llinas & Welsh, 1993), which in the case of cleaners, would modulate the response to novel clients (new partnerships).

On the other hand, in situations where cleaners were either prevented from interacting (group D—client inside the aquarium) or were less interested to interact (group E—in contact with a ball), the brain stem DOPAC levels and DOPAC/DA ratios increased. These results were probably associated to locomotor activity, for either moving around the client aquaria or just moving naturally (with no specific social purpose) in the control condition. For instance, in rainbow trout interacting for a 24 h period, during the establishment of dyadic dominant-subordinate relationships, DOPAC/DA ratios were observed to rise at the brain stem and hypothalamus of subordinates (fight losers) compared to dominants (fight winners) and controls (Øverli, Harris & Winberg, 1999). On the other hand, the levels of DA of cleaners that could interact fully with clients decreased at the diencephalon and optic tectum, two regions that otherwise have been positively associated with zebrafishs’ overt aggression (i.e., bites) during mirror fights (Teles et al., 2013).

### Serotoninergic change in accordance to social context

The diencephalons’ 5-HIAA/5-HT ratios increased in cleaners prevented to interact with clients and at the forebrain of cleaners prevented to interact with conspecifics, possibly through the activation of the posterior tuberculum/hypothalamic 5-HT neuronal populations. Considering that cleaners could not respond (aggressively or not) when introduced to individuals inside other aquaria, the activation of these areas may be a consequence of the inability to interact or rather, the impossibility to engage in contact with the client or conspecific in question.

The 5-HT levels decreased in cleaners in contact with clients, at the forebrain, diencephalon and optic tectum, and so did the 5-HIAA levels at the diencephalon and optic tectum. In nature, 5-HT exogenous increase, propels cleaners to seek and interact with clients, contrarily to the antagonists’ effects (Paula et al., 2015). Here, because we observed the effect after the actual interactive engagement, it was logical to expect a follow-up decrease of levels of 5-HT and 5-HIAA at specific regions of interest. Moreover, it is in contest situations (fighting) that 5-HT levels seem to increase at the telencephalon with the activation in the superior raphe projections (Teles et al., 2013), which is contrary to the welfare-like appraisal, demonstrated by clients during cleaning activities (Soares et al., 2011), and assumes: a dual absence of a stressor-like stimulus, and activation by the hypothalamic-pituitary-interrenal axis. Similarly, the 5-HTergic activity in the diencephalon represents the stimulation of the posterior tuberculum/hypothalamic 5-HT neuronal populations which again have been positively correlated with overt aggression (Teles et al., 2013).

Interestingly, we found increases in 5-HT and 5-HIAA levels at the optic tectum of cleaners interacting with conspecifics, in the control condition and amongst those prevented from engaging with clients (groups B and C, Fig. 1). These trends were not surprising considering that, for instance, in “losing” zebrafish (individuals that in a situation of direct confrontation are unable to overcome their partner), 5-HTergic activity was higher in the optic tectum (Kawato & Gomi, 1992). The optic tectum’ 5-HTergic fibers seem to originate from 5-HTergic neurons of the pretectal cluster (Kaslin & Panula, 2001), and have been implicated in the regulation of visual and motor behaviour (Bally-Cuif & Vernier, 2010), which may underline the elevated levels of activity reported by cleaners in the wild (cleaners interact with dozens of clients daily, see the introductory section, Bshary & Côté, 2008).

## Concluding Remarks

**Figure 3:**
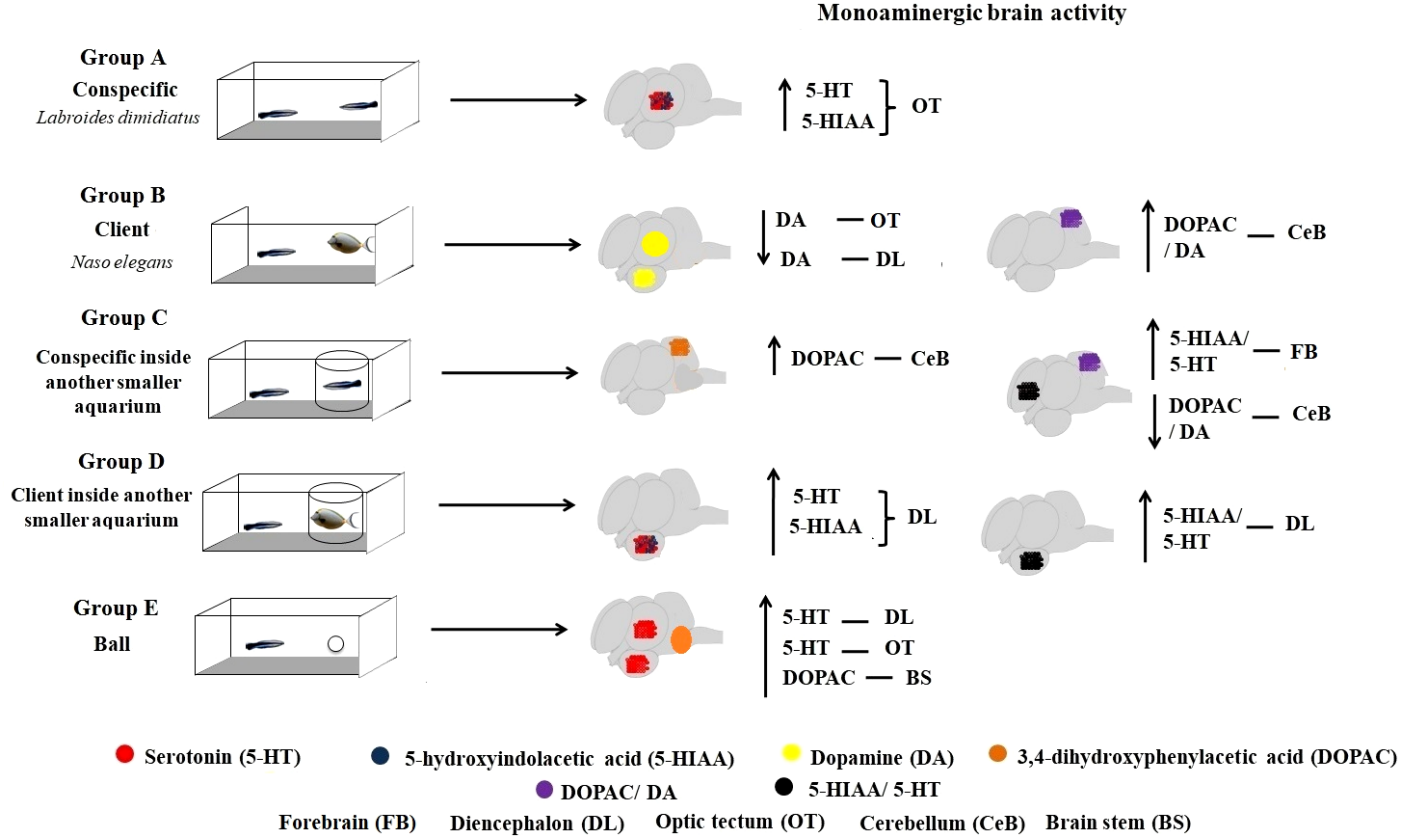
Monoaminergic brain activity in different social contexts.

Cleaners are active seekers of clients, either by approaching or chasing clients when these are just passing by, or simply by “dancing” up and down (publicity displays) which contribute to increase clients’ willingness to visit cleaning stations (Bshary & Côté, 2008). The role of 5-HTergic regulation in increasing cleaners’ propensity to interact has been crucially demonstrated (see Paula et al., 2015). Here, these 5-HTergic pathways were discovered to mainly change at the diencephalon responding to the absence of physical contact with clients while the DAergic function was mostly observed to respond at the cerebellum, when cleaners were in full contact with clients. Our results are the first demonstration of relevant brain (region-specific) monoaminergic change in cleaner fish, occurring in response to different mutualistic and conspecific contexts (see Fig. 3). These monoaminergic pathways should underlie cleaners drive to seek clients and conspecifics in nature, and to engage in physical (cleaning) contact with partners.

##  Supplemental Information

10.7717/peerj.4830/supp-1 Figure S1 Schematic drawing of the experimental groups (1) *Labroides dimidiatus* were distributed in five treatments: group A (conspecific, *L. dimidiatus*); group B (client, *N. elegans*); group C (conspecific inside another smaller aquarium); group D (client inside another smaller aquarium); and group E (white ball). (2) *Labroides dimidiatus* were separate brain regions with stereoscopic. (3) Brain regions were analysed the levels of dopamine and serotonin.

10.7717/peerj.4830/supp-2 Supplemental Information 1 Data for dopamine

10.7717/peerj.4830/supp-3 Supplemental Information 2 Data for behaviour

10.7717/peerj.4830/supp-4 Supplemental Information 3 Data for serotonine

10.7717/peerj.4830/supp-5 Supplemental Information 4 Data for individuals

## Additional Information and Declarations

### Data Availability

The following information was supplied regarding data availability: The raw data has been supplied as Supplemental Files.
