# Supplementary figures and images for: The variable monoaminergic outcomes of cleaner fish brains when facing different social and mutualistic contexts
Source: PeerJ. 2018 May 24;6:e4830. doi: 10.7717/peerj.4830 (PMC5971103; doi:10.7717/peerj.4830)

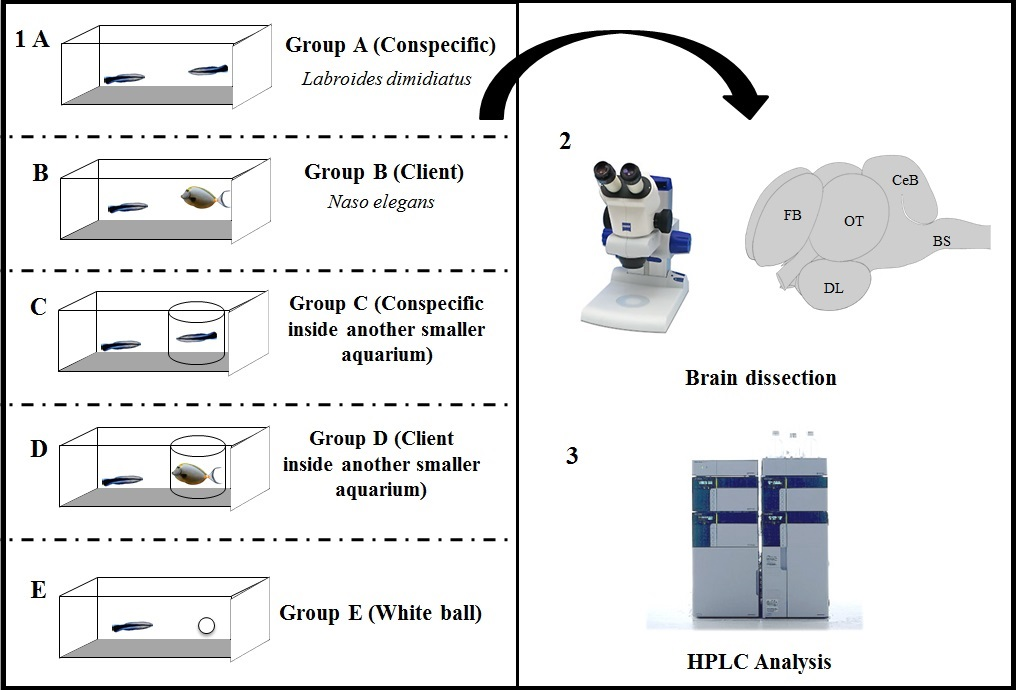

Supplement: Figure S1 — (1) Labroides dimidiatus were distributed in five treatments: group A (conspecific, L. dimidiatus); group B (client, N. elegans); group C (conspecific inside another smaller aquarium); group D (client inside another smaller aquarium); and group E (white ball). (2) Labroides dimidiatus were separate brain regions with stereoscopic. (3) Brain regions were analysed the levels of dopamine and serotonin. [file peerj-06-4830-s001.png]
